# Gender differences in healthy life years within the EU: an exploration of the “health–survival” paradox

**DOI:** 10.1007/s00038-012-0361-1

**Published:** 2012-05-22

**Authors:** Herman Van Oyen, Wilma Nusselder, Carol Jagger, Petra Kolip, Emmanuelle Cambois, Jean-Marie Robine

**Affiliations:** 1 Directorate of Public Health and Surveillance, Scientific Institute of Public Health, J. Wytsmanstraat 14, 1050 Brussels, Belgium; 2 Erasmus MC, University Medical Center Rotterdam, Rotterdam, The Netherlands; 3 Newcastle University, Newcastle, UK; 4 University of Bielefeld, Bielefeld, Germany; 5 French Institute for Demographic Studies, INED, Paris, France; 6 French Institute of Health and Medical Research, INSERM, Montpellier, France

**Keywords:** Europe, Gender, Health expectancy, Health inequality, Healthy life years, Health–survival paradox

## Abstract

**Objectives:**

To evaluated the female–male health–survival paradox by estimating the contribution of women’s mortality advantage versus women’s disability disadvantage.

**Methods:**

Disability prevalence was measured from the 2006 Survey on Income and Living Conditions in 25 European countries. Disability prevalence was applied to life tables to estimate healthy life years (HLY) at age 15. Gender differences in HLY were split into two parts: that due to gender inequality in mortality and that due to gender inequality in disability. The relationship between women’s mortality advantage or disability disadvantage and the level of population health between countries was analysed using random-effects meta-regression.

**Results:**

Women’s mortality advantage contributes to more HLY in women; women’s higher prevalence of disability reduces the difference in HLY. In populations with high life expectancy women’s advantage in HLY was small or even a men’s advantage was found. In populations with lower life expectancy, the hardship among men is already evident at young ages.

**Conclusions:**

The results suggest that the health–survival paradox is a function of the level of population health, dependent on modifiable factors.

## Introduction

Life expectancy in women is higher than in men. Although women live longer in nearly all countries of the world (Barford et al. 2006) the gender gap in life expectancy has narrowed during the last decades of the twentieth century in most but not all European countries (Van Oyen et al. 2010). Though several biological hypotheses have been proposed (Austad 2006), the dynamics of the gender differences in mortality suggest that its determinants cannot be purely biological, but are also dependent on modifiable psycho-social and lifestyle factors (Barford et al. 2006; Gjonça et al. 2005). In developed countries smoking has been considered as one of the main causes of the gender difference in mortality (Jacobsen et al. 2008; Leon 2011; Pampel 2003; Payne 2004; Preston and Wang 2006). Within Europe, smoking accounts for 40–60 % of the mortality difference by gender, while alcohol contributes to 10–30 % of the gender gap (McCartney et al. 2011).

In many countries the mortality advantage of women is balanced by a disability disadvantage (Case and Paxson 2005). This contrast is called the female–male health–survival paradox (Oksuzyan et al. 2008). The proposed explanations for the health–survival paradox are rooted in biological, social, and psychological interpretations (Oksuzyan et al. 2008). Although selection and information bias from gender differences in participation in surveys and reporting cannot be excluded, the contribution of both biases to the health–survival paradox is considered to be small (Oksuzyan et al. 2009).

To better understand the dynamics of population health and especially, the female–male health–survival paradox, the use of composite health indicators, such as health expectancies, has been proposed (Nusselder et al. 2010b). Health expectancies are summary measures of population health bringing together data on both the quantity and the quality of life (Robine 2006). They are considered to be important population health outcome measures (Stiefel et al. 2010). Health expectancies, predominantly disability-free life expectancy (DFLE), are available for many countries worldwide (Robine et al. 2003). Within the European Union, it was decided to estimate DFLE based on a measure of long-term activity limitations (Van Oyen et al. 2006), the healthy life years (HLY). Similar to life expectancy, HLY at a given age corresponds to the average life span free of activity limitation. The average life span with activity limitation is called unhealthy life years (ULY).

Gender difference in both HLY and ULY, can be split into two components: (1) the difference due to inequality in age-specific mortality rates (“mortality effect”: gender difference in life expectancy without or with activity limitations due to differential mortality); and (2) difference due to gender inequality in age-specific prevalence of activity limitations (“disability effect”: difference in life expectancy without or with activity limitations because of differences in the prevalence of activity limitations).

The current paper aims to better understand the health–survival paradox within the EU by examining the contribution of women’s mortality advantage versus the disability disadvantage, and their differences between countries with better and worse population health. More specifically, we explore the following questions:

1. Does the mortality advantage and/or disability disadvantage of women vary between countries with high versus low life expectancy? We expect that in populations with high life expectancy, the gender gap in HLY is smaller because of a combination of a smaller mortality advantage and a larger disability disadvantage in females.
2. Is there a shift in the concentration of the mortality and disability effects on the gender gap in HLY towards older age groups (50 years and above) when indicators of population health (e.g., having a longer life, a longer life without activity limitations or a shorter life with activity limitations) are improving? We hypothesize that in countries with better scores on the population health indicators, the effects of both mortality and disability on the gender gap in HLY is more concentrated in older age groups.

The added value of the paper is to study the health–survival paradox through HLY, which combines both health and survival.

## Methods

### Data

We used EU member states specific data of the European Health Expectancy Monitoring Unit Information System (http://www.ehemu.eu): age and sex-specific data on (1) number of deaths (2006); (2) population (2006, 2007); and (3) prevalence of activity limitations (number of persons with activity limitations and the total number in the sample) from the 2006 Statistics of Living and Income Survey (SILC). The SILC is an EU-wide survey, initiated in 2005. A description of the survey can be found in “http://circa.europa.eu/public/irc/dsis/eusilc.library”. The SILC survey population consists of nationally representative probabilistic samples from community dwelling populations. The 2006 SILC survey covers a total of 375,243 participants of age 16 years and above. Overall response rate averaged over countries is about 20 % but with large between countries variations (from 95 % in Cyprus to 60 % in Denmark and Belgium) (Eurostat 2009).

### Activity limitations

The SILC contains the Minimum European Health Module (Robine and Jagger 2003), which includes a disability measure, the global activity limitation indicator (GALI). The GALI (“For at least the last 6 months, have you been limited because of a health problem in activities people usually do?”) aims to capture long-term limitation in usual activities, caused by ill-health (Van Oyen et al. 2006) and provides the health status information to calculate HLY. The validity and the reliability of the GALI have been documented (Cox et al. 2009; Jagger et al. 2010; Van Oyen et al. 2006).

### Statistical methods

HLY at age 15 was calculated using the Sullivan method, which integrates age-specific disability prevalence into the life table (Jagger et al. 2007; Sullivan 1971). ULY are calculated as the difference between life expectancy (LE) and HLY.

To estimate the contribution of the mortality and disability effects to the gender differences (females − males) in HLY and ULY, a decomposition methodology was used (Nusselder et al. 2005; Nusselder and Looman 2004). This method is an extension of the decomposition techniques used in mortality research (Arriaga 1984) to assess the contribution of age or specific diseases to differences in LE. Gender inequalities in LE reflect differences in mortality rates only. Gender differences in HLY or ULY are a result of differences in mortality combined with differences in the prevalence of activity limitations. The decomposition method allows the estimation of the percentage of mortality or disability effects of the gender difference in HLY (or in ULY) that are due to differences in specific age groups (e.g., younger ages (15–49 years) or older ages (50+ years)). Calculations were done using R. A statistical description of the decomposition methods including a manual for the R-macro has been described http://www.eurohex.eu/pdf/Reports_2010/2010TR7.1_Decomposition%20tools.pdf (Nusselder et al. 2010a). The variance and 95 % CI around the mortality and disability effects were estimated by a bootstrap procedure with 1,000 resamples (Efron and Tibshirani 1993) with the assumption that the number of age-specific deaths followed a Poisson distribution, and the number of persons with activity limitations resampled within the sample size of the age-specific survey groups followed a binomial distribution. The 2.5 and 97.5 percentile of the bootstrap distribution defined the 95 % CI boundaries.

To evaluate the health–survival paradox we investigate the relationship between the mortality advantage or the disability disadvantage that women experience over men and the duration and/or the gender gap in the duration of total, healthy or unhealthy life using random-effects meta-regression models in STATA-10 (Sutton and Abrams 2001). In contrast to ordinary regression models, these models account for the uncertainty around the country-specific mortality or disability effect.

We use three univariable models (model Type 1) each assessing the relationship of the mortality effect of the gender difference in HLY (dependent variable) with an independent variable representing the overall length of life (women’s LE, men’s LE and gender difference in LE). Model Type 2 is multivariable, adjusting the gender difference in LE for women’s LE to account for the association between gender difference in LE and longer life (Van Oyen et al. 2010). A similar modelling process (Type 1 and Type 2) is adopted for assessing the relationship between the mortality effect of the gender difference in HLY and overall HLY (women’s HLY, men’s HLY and gender difference in HLY) and then overall ULY. Finally, the whole is repeated for assessing (1) the relationship of the disability effect of the gender difference in HLY with the overall length of life, of healthy life and of unhealthy life and (2) the relationship of the mortality or disability effect of the gender difference in ULY with the overall length of life, of healthy life and of unhealthy life. We present the univariable associations by line graphs of the fitted values, with the estimates from each member state represented by circles, the circle sizes depending on the precision of each estimate (the inverse of its within-country variance), which is the weight given to each country in the model. Similar models were used when the dependent variable was the relative contribution (%) of older age (50+ years) to the mortality and disability effect of the gender difference in HLY or in ULY.

## Results

**Fig. 1:**
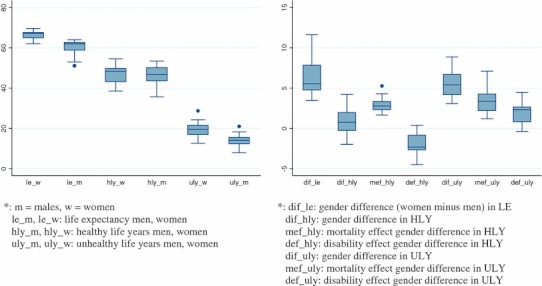
Distribution (in years) of life expectancy (le_*), healthy and unhealthy live years (hly_*, uly_*), the gender differences (female − male; dif_*) and the decomposition indicators by kind of effect (mortality (mef_*) and disability (def_*) effect) at age 15 years, EU-25, 2006

Tables 1 and 2 provide the estimates of the different health expectancy indicators, the gender differences (female − male) and the decomposition by type of effect at age 15 years. Data are summarized by boxplots (Fig. 1). Women’s LE at age 15 years always exceeds male LE, but LE varies substantially across countries as does the gender difference which varies from 3.5 to 11.6 years.

**Table 1:** Healthy (HLY), unhealthy life years (ULY), Life Expectancy (LE) and the  % of remaining life in good health (%HLY) at age 15 in European Member States and in the European Union (EU-25), 2006

Country	Males				Females			
	HLY	ULY	LE	%HLY/LE	HLY	ULY	LE	%HLY/LE
Austria	44.23 (43.52; 44.84)	18.24 (17.63; 18.96)	62.48 (62.35; 62.61)	70.8 (69.66; 71.76)	46.48 (45.81; 47.20)	21.51 (20.79; 22.20)	67.99 (67.88; 68.11)	68.36 (67.36; 69.43)
Belgium	48.55 (47.90; 49.29)	13.40 (12.65; 14.07)	61.96 (61.84; 62.06)	78.37 (77.29; 79.58)	48.67 (47.83; 49.44)	18.84 (18.05; 19.64)	67.50 (67.40; 67.60)	72.10 (70.91; 73.27)
Cyprus	49.95 (49.13; 50.82)	14.04 (13.18; 14.85)	63.99 (63.58; 64.37)	78.05 (76.82; 79.37)	48.59 (47.80; 49.45)	18.87 (18.01; 19.73)	67.47 (67.10; 67.84)	72.03 (70.81; 73.24)
Czech Republic	43.71 (43.09; 44.28)	15.09 (14.53; 15.72)	58.8 (58.70; 58.91)	74.34 (73.27; 75.30)	45.68 (45.06; 46.27)	19.41 (18.80; 20.05)	65.10 (65.00; 65.19)	70.18 (69.20; 71.10)
Denmark	53.40 (52.60; 54.13)	8.03 (7.29; 8.80)	61.43 (61.28; 61.57)	86.93 (85.67; 88.12)	52.95 (52.02; 53.92)	13.03 (12.05; 14.00)	65.98 (65.85; 66.12)	80.25 (78.81; 81.72)
Estonia	35.69 (35.06; 36.27)	17.28 (16.69; 17.88)	52.96 (52.65; 53.29)	67.38 (66.26; 68.45)	39.87 (39.26; 40.51)	24.00 (23.33; 24.61)	63.86 (63.60; 64.12)	62.42 (61.49; 63.43)
Finland	39.21 (38.36; 40.03)	21.06 (20.24; 21.92)	60.27 (60.14; 60.42)	65.06 (63.64; 66.42)	39.52 (38.55; 40.45)	28.69 (27.79; 29.63)	68.21 (68.08; ; 68.37)	57.94 (56.53; 59.29)
France	48.55 (48.03; 49.02)	14.12 (13.64; 14.63)	62.67 (62.62; 62.71)	77.47 (76.65; 78.23)	49.79 (49.24; 50.38)	19.82 (19.23; 20.38)	69.61 (69.56; 69.66)	71.52 (70.72; 72.38)
Germany	44.18 (43.66; 44.66)	18.28 (17.78; 18.80)	62.45 (62.41; 62.50)	70.74 (69.90; 71.52)	43.94 (43.44; 44.52)	23.70 (23.13; 24.21)	67.64 (67.61; 67.68)	64.96 (64.20; 65.80)
Greece	51.76 (51.21; 52.30)	10.69 (10.15; 11.23)	62.45 (62.35; 62.56)	82.88 (82.02; 83.74)	53.43 (52.82; 54.06)	13.80 (13.17; 14.39)	67.23 (67.14; 67.32)	79.48 (78.60; 80.40)
Hungary	40.11 (39.62; 40.59)	14.67 (14.19; 15.15)	54.77 (54.67; 54.88)	73.22 (72.34; 74.09)	42.71 (42.23; 43.19)	20.49 (20.01; 20.98)	63.20 (63.10; 63.31)	67.58 (66.82; 68.33)
Ireland	48.97 (48.34; 49.60)	13.81 (13.19; 14.45)	62.78 (62.61; 62.95)	78.00 (76.98; 79.00)	50.45 (49.77; 51.13)	16.96 (16.24; 17.66)	67.41 (67.22; 67.59)	74.84 (73.82; 75.88)
Italy	50.18 (49.85; 50.49)	13.59 (13.27; 13.91)	63.77 (63.72; 63.81)	78.69 (78.17; 79.19)	49.74 (49.38; 50.07)	19.55 (19.25; 19.91)	69.29 (69.25; 69.33)	71.78 (71.26; 72.24)
Latvia	36.71 (36.08; 37.41)	14.53 (13.86; 15.19)	51.25 (51.00; 51.48)	71.64 (70.42; 72.93)	38.63 (37.92; 39.36)	23.42 (22.71; 24.13)	62.06 (61.85; 62.26)	62.26 (61.16; 63.41)
Lithuania	38.48 (37.88; 39.08)	12.54 (11.93; 13.12)	51.01 (50.82; 51.22)	75.42 (74.29; 76.59)	42.15 (41.53; 42.80)	20.51 (19.83; 21.13)	62.66 (62.49; 62.84)	67.26 (66.27; 68.33)
Luxembourg	46.78 (45.83; 47.70)	15.14 (14.26; 16.17)	61.92 (61.39; 62.47)	75.55 (74.04; 76.95)	47.55 (46.43; 48.63)	19.41 (18.34; 20.53)	66.95 (66.46; 67.47)	71.01 (69.37; 72.60)
Malta	53.49 (52.69; 54.29)	8.81 (8.12; 9.53)	62.30 (61.81; 62.86)	85.87 (84.74; 86.96)	54.5 (53.70; 55.29)	12.63 (11.85; 13.43)	67.13 (66.67; 67.62)	81.18 (80.08; 82.32)
Netherlands	50.90 (50.06; 51.69)	12.19 (11.40; 13.02)	63.09 (63.01; 63.17)	80.68 (79.37; 81.93)	48.91 (48.03; 49.73)	18.41 (17.58; 19.29)	67.32 (67.25; 67.40)	72.66 (71.34; 73.89)
Poland	44.09 (43.74; 44.43)	12.39 (12.06; 12.74)	56.48 (56.41; 56.54)	78.06 (77.45; 78.64)	48.33 (47.97; 48.73)	16.74 (16.34; 17.11)	65.07 (65.01; 65.12)	74.28 (73.71; 74.89)
Portugal	45.21 (44.59; 45.86)	15.71 (15.04; 16.31)	60.92 (60.81; 61.02)	74.21 (73.25; 75.30)	43.24 (42.49; 43.99)	24.26 (23.48; 24.99)	67.49 (67.40; 67.60)	64.06 (62.96; 65.19)
Slovakia	40.49 (39.90; 41.07)	15.58 (15.03; 16.20)	56.08 (55.92; 56.23)	72.21 (71.13; 73.22)	40.48 (39.84; 41.08)	23.43 (22.82; 24.06)	63.91 (63.78; 64.06)	63.34 (62.34; 64.27)
Slovenia	43.68 (42.88; 44.47)	15.95 (15.18; 16.77)	59.63 (59.39; 59.88)	73.25 (71.94; 74.53)	47.35 (46.49; 48.19)	19.77 (18.91; 20.61)	67.11 (66.89; 67.35)	70.55 (69.28; 71.79)
Spain	49.38 (48.97; 49.79)	13.67 (13.26; 14.07)	63.05 (62.99; 63.10)	78.32 (77.68; 78.98)	48.98 (48.46; 49.50)	20.67 (20.15; 21.17)	69.65 (69.59; 69.70)	70.33 (69.60; 71.08)
Sweden	52.58 (51.73; 53.44)	11.48 (10.61; 12.33)	64.06 (63.95; 64.17)	82.08 (80.75; 83.45)	52.93 (51.97; 53.90)	15.30 (14.35; 16.27)	68.23 (68.13; 68.34)	77.57 (76.17; 78.97)
United Kingdom	50.71 (50.20; 51.19)	12.07 (11.61; 12.58)	62.78 (62.73; 62.83)	80.77 (79.98; 81.52)	50.80 (50.19; 51.38)	16.27 (15.70; 16.87)	67.07 (67.03; 67.12)	75.74 (74.85; 76.60)
EU-25	47.36 (47.25; 47.48)	14.30 (14.18; 14.42)	61.66 (61.65; 61.68)	76.81 (76.62; 77.00)	47.91 (47.79; 48.05)	19.76 (19.63; 19.88)	67.67 (67.66; 67.69)	70.70 (70.62; 71.00)

HLY and ULY are estimated using the Sullivan method; member states gender and age-specific mortality and morbidity (SILC 2006) data. Values in parentheses are 95 % confidence interval

**Table 2:** Decomposition of the gender difference (females − males) in healthy life years (HLY), unhealthy life years (ULY) and total life expectancy (LE) at age 15 by type of effect (mortality or disability) in European Member States and in the European Union (EU-25), 2006

Country	HLY			ULY			LE		
	Difference	Mortality effect	Disability effect	Difference	Mortality effect	Disability effect	Difference	Mortality effect	Disability effect
Austria	2.25 (1.31; 3.24)	2.13 (2.00; 2.27)	0.11 (−0.87; 1.11)	3.27 (2.26; 4.17)	3.38 (3.23; 3.53)	−0.11 (−1.11; 0.87)	5.52 (5.34; 5.68)	5.52 (5.34; 5.68)	0
Belgium	0.11 (−0.98; 1.11)	2.76 (2.58; 2.95)	−2.65 (−3.78; −1.62)	5.43 (4.40; 6.56)	2.79 (2.61; 2.97)	2.65 (1.62; 3.78)	5.55 (5.40; 5.69)	5.55 (5.40; 5.69)	0
Cyprus	−1.36 (−2.51; −0.24)	1.68 (1.40; 1.97)	−3.03 (−4.21; −1.91)	4.83 (3.69; 5.99)	1.80 (1.49; 2.13)	3.03 (1.91; 4.21)	3.47 (2.93; 4.02)	3.47 (2.93; 4.02)	0
Czech Republic	1.97 (1.12; 2.86)	2.81 (2.68; 2.94)	−0.84 (−1.68; 0.06)	4.32 (3.43; 5.16)	3.48 (3.35; 3.62)	0.84 (−0.06; 1.68)	6.29 (6.15; 6.42)	6.29 (6.15; 6.42)	0
Denmark	−0.45 (−1.70; 0.80)	3.35 (3.12; 3.57)	−3.8 (−5.00; −2.54)	5.00 (3.72; 6.20)	1.20 (1.04; 1.38)	3.80 (2.54; 5.00)	4.55 (4.36; 4.74)	4.55 (4.36; 4.74)	0
Estonia	4.18 (3.38; 5.04)	3.8 (3.57; 4.07)	0.37 (−0.48; 1.27)	6.72 (5.81; 7.57)	7.10 (6.80; 7.44)	−0.37 (−1.27; 0.48)	10.90 (10.50; 11.32)	10.90 (10.50; 11.32)	0
Finland	0.31 (−0.91; 1.55)	2.78 (2.55; 3.06)	−2.47 (−3.72; −1.24)	7.63 (6.38; 8.88)	5.16 (4.88; 5.43)	2.47 (1.24; 3.72)	7.94 (7.74; 8.16)	7.94 (7.74; 8.16)	0
France	1.24 (0.50; 2.04)	2.97 (2.83; 3.11)	−1.73 (−2.49; −0.94)	5.70 (4.92; 6.45)	3.98 (3.83; 4.12)	1.73 (0.94; 2.49)	6.95 (6.87; 7.01)	6.95 (6.87; 7.01)	0
Germany	−0.24 (−0.91; 0.53)	2.08 (1.99; 2.18)	−2.32 (−3.01; −1.52)	5.43 (4.66; 6.11)	3.11 (3.01; 3.20)	2.32 (1.52; 3.01)	5.19 (5.14; 5.24)	5.19 (5.14; 5.24)	0
Greece	1.67 (0.85; 2.48)	2.91 (2.80; 3.02)	−1.24 (−2.07; −0.44)	3.1 (2.29; 3.92)	1.86 (1.77; 1.96)	1.24 (0.44; 2.07)	4.77 (4.63; 4.92)	4.77 (4.63; 4.92)	0
Hungary	2.60 (1.90; 3.26)	3.39 (3.26; 3.53)	−0.79 (−1.50; −0.11)	5.82 (5.16; 6.52)	5.03 (4.88; 5.19)	0.79 (0.11; 1.50)	8.43 (8.27; 8.58)	8.43 (8.27; 8.58)	0
Ireland	1.48 (0.54; 2.48)	2.33 (2.17; 2.50)	−0.84 (−1.77; 0.16)	3.14 (2.11; 4.08)	2.30 (2.14; 2.47)	0.84 (−0.16; 1.77)	4.63 (4.38; 4.90)	4.63 (4.38; 4.90)	0
Italy	−0.44 (−0.92; 0.07)	2.27 (2.20; 2.34)	−2.71 (−3.19; −2.21)	5.96 (5.47; 6.44)	3.25 (3.18; 3.32)	2.71 (2.21; 3.19)	5.53 (5.46; 5.58)	5.53 (5.46; 5.58)	0
Latvia	1.92 (0.92; 2.90)	4.27 (4.03; 4.49)	−2.35 (−3.37; −1.38)	8.89 (7.92; 9.84)	6.54 (6.28; 6.80)	2.35 (1.38; 3.37)	10.81 (10.51; 11.11)	10.81 (10.51; 11.11)	0
Lithuania	3.67 (2.81; 4.56)	5.27 (5.05; 5.50)	−1.60 (−2.42; −0.72)	7.98 (7.13; 8.83)	6.38 (6.14; 6.60)	1.6 (0.72; 2.42)	11.65 (11.37; 11.92)	11.65 (11.37; 11.92)	0
Luxembourg	0.77 (−0.70; 2.13)	2.33 (1.90; 2.77)	−1.56 (−2.97; −0.23)	4.27 (2.80; 5.61)	2.71 (2.26; 3.16)	1.56 (0.23; 2.97)	5.04 (4.33; 5.76)	5.04 (4.33; 5.76)	0
Malta	1.01 (−0.02; 2.16)	2.62 (2.19; 3.08)	−1.61 (−2.60; −0.54)	3.83 (2.81; 4.84)	2.22 (1.84; 2.58)	1.61 (0.54; 2.60)	4.84 (4.14; 5.58)	4.84 (4.14; 5.58)	0
Netherlands	−1.98 (−3.12; −0.79)	2.18 (2.00; 2.37)	−4.17 (−5.33; −2.99)	6.22 (5.03; 7.40)	2.05 (1.87; 2.22)	4.17 (2.99; 5.33)	4.23 (4.12; 4.34)	4.23 (4.12; 4.34)	0
Poland	4.24 (3.78; 4.78)	4.31 (4.19; 4.43)	−0.06 (−0.55; 0.47)	4.34 (3.79; 4.82)	4.28 (4.16; 4.39)	0.06 (−0.47; 0.55)	8.59 (8.51; 8.66)	8.59 (8.51; 8.66)	0
Portugal	−1.97 (−2.94; −1.02)	2.52 (2.38; 2.65)	−4.49 (−5.47; −3.58)	8.55 (7.61; 9.52)	4.05 (3.90; 4.21)	4.49 (3.58; 5.47)	6.57 (6.44; 6.73)	6.57 (6.44; 6.73)	0
Slovakia	−0.01 (−0.82; 0.85)	2.65 (2.47; 2.83)	−2.66 (−3.48; −1.76)	7.85 (6.99; 8.65)	5.19 (4.99; 5.40)	2.66 (1.76; 3.48)	7.84 (7.64; 8.04)	7.84 (7.64; 8.04)	0
Slovenia	3.67 (2.52; 4.85)	3.83 (3.54; 4.12)	−0.16 (−1.32; 1.08)	3.81 (2.65; 4.97)	3.65 (3.39; 3.92)	0.16 (−1.08; 1.32)	7.48 (7.14; 7.84)	7.48 (7.14; 7.84)	0
Spain	−0.40 (−1.08; 0.20)	3.22 (3.12; 3.33)	−3.63 (−4.28; −3.02)	7.00 (6.40; 7.67)	3.37 (3.26; 3.48)	3.63 (3.02; 4.28)	6.59 (6.52; 6.67)	6.59 (6.52; 6.67)	0
Sweden	0.35 (−0.89; 1.64)	2.83 (2.65; 3.03)	−2.48 (−3.76; −1.21)	3.82 (2.56; 5.07)	1.34 (1.17; 1.50)	2.48 (1.21; 3.76)	4.17 (4.02; 4.33)	4.17 (4.02; 4.33)	0
United Kingdom	0.09 (−0.66; 0.81)	2.47 (2.39; 2.56)	−2.38 (−3.12; −1.66)	4.20 (3.49; 4.95)	1.82 (1.74; 1.90)	2.38 (1.66; 3.12)	4.29 (4.23; 4.36)	4.29 (4.23; 4.36)	0
EU-25	0.55 (0.37; 0.74)	2.86 (2.83; 2.89)	−2.31 (−2.50; −2.12)	5.46 (5.27; 5.64)	3.15 (3.12; 3.17)	2.31 (2.12; 2.50)	6.01 (5.98; 6.03)	6.01 (5.98; 6.03)	0

Values in parentheses are 95 % confidence interval

The median gender difference in HLY (0.8 years) is less than that for LE (5.6 years). The variation in the gender difference in HLY [interquartile range (IQR): 2.4, range: 6.2] is also smaller than that for LE (IQR: 3.2, range: 8.2). In 7 countries (Cyprus, Denmark, Germany, Italy, the Netherlands, Portugal and Spain), men at age 15 can expect to live more years without activity limitations than women, the gender difference in HLY reaching statistical significance in 3 of these countries (Cyprus, the Netherlands and Portugal). In all countries, women live more years with activity limitations than men, the median gender difference in ULY being 5.4 years (IQR 2.8, range 5.8).

### Decomposition of the gender difference in HLY

The gender difference is HLY is the sum of two opposing forces. Women’s mortality advantage increases the HLY gender difference, while women’s disability disadvantage reduces the HLY gender difference. In all countries, the value of the mortality effect is positive (Table 2) meaning that women’s mortality advantage over men contributes to more HLY in women. On average this amounts to 2.8 HLY (IQR 1.04, range 3.6 years) with the highest value (5.3 years) in Lithuania as an outlier (Fig. 1). In all but two countries (Austria and Estonia), the disability effect on HLY is negative, meaning that the higher prevalence of activity limitations in women reduces the gender difference in HLY. The size of these two opposing effects varies according to the country: women’s disability disadvantage cancels out women’s mortality advantage in seven countries so that in these countries men have a longer expectation of life without activity limitations.

**Fig. 2:**
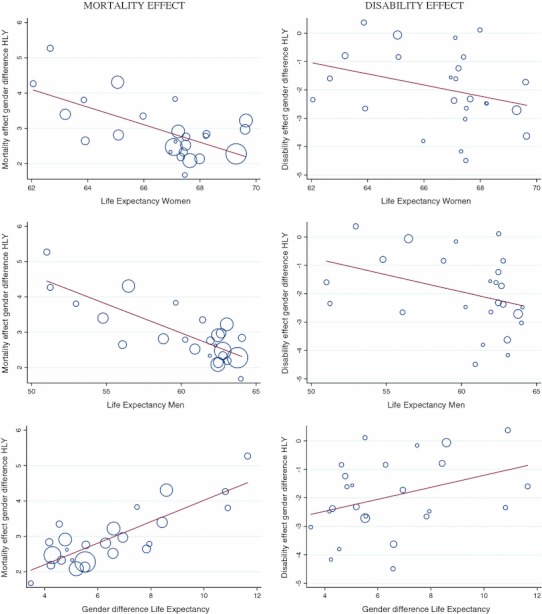
Association between the life expectancy and decomposition indicators by kind of effect of the gender differences (females − males) in healthy life years (HLY) at age 15 years, EU-25, 2006. Meta-regression (univariable) line graph of fitted values. *Circles* represent the estimate (mortality or disability effect) from each country, with circle size depending on the inverse of the within-country variance

Figure 2 shows the results of six different univariable meta-regression analyses. In the left column, the contribution of mortality to the gender gap in HLY is presented as a function of women’s LE, men’s LE and gender difference in LE. The right column presents the disability effect. When women’s or men’s LE is larger, the HLY gender gap is reduced mainly because women’s mortality advantage is smaller but also to a lesser extent due to a larger disability disadvantage although the latter did not reach statistical significance (Table 3a). For example, a 1-year increase in women’s LE goes along with a 0.25-year decrease of the mortality effect of the HLY gender gap (coefficient_women’s LE_: −0.25, Table 3a) while the effect of women’s disability disadvantage is larger (coefficient_women’s LE_: −0.20, Table 3a). For every increase of 1 year in women’s LE, the HLY gender gap is reduced by 0.45 years [(−0.25) + (−0.20)].

**Table 3:** Association of the mortality and disability effect of the gender differences in years (females − males) in healthy life years (HLY) and in unhealthy life years (ULY) with the life expectancy (LE), HLY and ULY at age 15 years, EU, 2006

Mortality effect						Disability effect					
Model Type 1 (9 univariable models)^a^			Model Type 2 (3 multivariable models)^b^			Model Type 1 (9 univariable models)			Model Type 2 (3 multivariable models)		
(a) Gender difference in HLY											
LE						LE					
1: LE men	−0.16^c^	**				1: LE men	−0.12				
2: LE women	−0.25	**	1: LE women	−0.05		2: LE women	−0.20		1: LE women	−0.08	
3: Gender difference LE	0.30	**	1: Gender difference LE	0.27	**	3: Gender difference LE	0.21		1: Gender difference LE	0.16	
HLY						HLY					
4: HLY men	−0.08	**				4: HLY men	−0.10				
5: HLY women	−0.06		2: HLY women	−0.03		5: HLY women	−0.03		2: HLY women	0.03	
6: Gender difference HLY	0.33	**	2: Gender difference HLY	0.31	**	6: Gender difference HLY	0.65	**	2: Gender difference HLY	0.67	**
ULY						ULY					
7: ULY men	−0.04					7: ULY men	0.10				
8: ULY women	0.01		3: ULY women	−0.08		8: ULY women	−0.01		3: ULY women	0.19	*
9: Gender difference ULY	0.16		3: Gender difference ULY	0.28	*	9: Gender difference ULY	−0.35	**	3: Gender difference ULY	−0.63	**
(b) Gender difference in ULY											
LE						LE					
1: LE men	−0.35	**				1: LE men	0.12				
2: LE women	−0.49	**	1: LE women	0.05		2: LE women	0.20		1: LE women	0.08	
3: Gender difference LE	0.70	**	1: Gender difference LE	0.73	**	3: Gender difference LE	−0.21		1: Gender difference LE	−0.16	
HLY						HLY					
4: HLY men	−0.29	**				4: HLY men	0.10	*			
5: HLY women	−0.31	**	2: HLY women	−0.28	**	5: HLY women	0.03		2: HLY women	−0.03	
6: Gender difference HLY	0.49	**	2: Gender difference HLY	0.32	**	6: Gender difference HLY	−0.65	**	2: Gender difference HLY	−0.67	**
ULY						ULY					
7: ULY men	0.29	**				7: ULY men	0.10				
8: ULY women	0.31	**	3: ULY women	0.19		8: ULY women	−0.01		3: ULY women	0.19	*
9: Gender difference ULY	0.65	**	3: Gender difference ULY	0.36	*	9: Gender difference ULY	−0.35	**	3: Gender difference ULY	−0.63	**

* Significant at *p* = 0.05; ** significant at *p* = 0.01

^a^Model Type 1: univariable meta-regression

^b^Model Type 2: multivariable meta-regression: the association of gender difference in the LE, HLY, or ULY with the mortality effect or disability effect (in years) of the gender difference in (a) HLY or in (b) ULY is adjusted for the duration of LE, HLY, or ULY among females

^c^Coefficient of the meta-regression indicating the change in the dependent variables (e.g. mortality effect or disability effect (in years) of the gender difference in (a) HLY or in (b) ULY) in function of the change in the independent variables [LE, HLY, or ULY, Gender difference (in LE, HLY, or ULY) in years]

The wider the gender gap in LE, the larger the gender difference in HLY due to a larger mortality women advantage (coefficient_LE_gender_gap_: 0.30), and a smaller women’s disability disadvantage (coefficient_LE_gender_gap_: 0.21). For every increase of 1 year in the gender gap in LE, the HLY gender gap increases by 0.51 years [(0.30) + (0.21)]. Only the mortality component of the HLY gender difference is significantly associated with the gender difference in LE and the association remains significant after adjustment for women’s LE.

When the health of populations improves (measured by a higher LE, higher HLY or a lower ULY), gender differences in mortality at older ages (50 years and above) contribute more proportionally to the mortality effect of the gender difference in HLY. For instance, for every increase of 1 year in women’s LE, the gender differences in the age group 50+ years contribute almost 3 % more to the mortality effect of the gender difference in HLY (coefficient_women’s LE_ is 2.96, Table 4a). On the other hand, when the gender difference in either LE, HLY or ULY is large, the percentage of the mortality effect on the gender difference in HLY that can be attributed to gender differences in mortality at older ages decreases (e.g., a decrease of 3.3 % for every increase of 1 year in the gender difference in LE). None of the associations of the relative contribution of older age to the disability effect on the gender difference in HLY were statistically significant.

**Table 4:** Association of relative contribution (%) of older age (50 years and above) to the mortality and disability effect of the gender differences (females − males) in healthy life years (HLY) and in unhealthy life years (ULY) with the life expectancy (LE), HLY and ULY at age 15 years, EU, 2006

The percent contribution of older age (50 years and above) to the mortality effect						The percent contribution of older age (50 years and above) to the disability effect			
Model Type 1 (9 univariable models)^a^			Model Type 2 (3 multivariable models)^b^			Model Type 1 (9 univariable models)		Model Type 2 (3 multivariable models)	
(a) Gender difference in HLY									
LE						LE			
1: LE men	1.86^c^	**				1: LE men	−0.38		
2: LE women	2.96	**	1: LE women	0.99		2: LE women	−1.87	1: LE women	−4.20
3: Gender difference LE	−3.30	**	1: Gender difference LE	−2.67	**	3: Gender difference LE	−0.44	1: Gender difference LE	−3.15
HLY						HLY			
4: HLY men	1.28	**				4: HLY men	1.34		
5: HLY women	1.32	**	2: HLY women	1.15	**	5: HLY women	3.97	2: HLY women	5.65
6: Gender difference HLY	−2.58	**	2: Gender difference HLY	−1.87	**	6: Gender difference HLY	14.74	2: Gender difference HLY	18.25
ULY						ULY			
7: ULY men	−0.78					7: ULY men	−5.24		
8: ULY women	−1.04	**	3: ULY women	−0.29		8: ULY women	−6.39	3: ULY women	−2.49
9: Gender difference ULY	−2.82	**	3: Gender difference ULY	−2.39		9: Gender difference ULY	−16.11	3: Gender difference ULY	−12.34
(b) Gender difference in ULY									
LE					LE				
1: LE men	1.18	**	1: LE men			1: LE men	−0.38	1: LE men	
2: LE women	2.03	**	2: LE women	1.08	**	2: LE women	−1.87	2: LE women	−4.20
3: Gender difference LE	−1.98	**	3: Gender difference LE	−1.28	**	3: Gender difference LE	−0.44	3: Gender difference LE	−3.15
HLY						HLY			
4: HLY men	0.74	**	4: HLY men			4: HLY men	1.34	4: HLY men	
5: HLY women	0.73	**	5: HLY women	0.61	**	5: HLY women	3.97	5: HLY women	5.65
6: Gender difference HLY	−1.70	**	6: Gender difference HLY	−1.32	**	6: Gender difference HLY	14.74	6: Gender difference HLY	18.25
ULY					ULY				
7: ULY men	−0.23		7: ULY men			7: ULY men	−5.24	7: ULY men	
8: ULY women	−0.45	**	8: ULY women	0.06		8: ULY women	−6.39	8: ULY women	−2.49
9: Gender difference ULY	−1.53	**	9: Gender difference ULY	−1.62	*	9: Gender difference ULY	−16.11	9: Gender difference ULY	−12.34

* Significant at *p* = 0.05; ** significant at *p* = 0.01

^a^Model Type 1: univariable meta-regression

^b^Model Type 2: multivariable meta-regression: the association of gender difference in the LE, HLY, or ULY with the percent contribution of older age (50 years and above) to the mortality effect or the disability effect (in  %) of the gender difference in (a) HLY or in (b) ULY is adjusted for the duration of LE, HLY, or ULY among females

^c^Coefficient of the meta-regression indicating the change in the dependent variables (the percent contribution of older age (50 years and above) to the mortality and disability effect of the gender difference in (a) HLY or in (b) ULY in function of the change in the independent variables (LE, HLY, ULY, Gender difference (LE, HLY, ULY) in years)

### Decomposition of the gender difference in ULY

A positive value of the ULY mortality effect indicates that women’s mortality advantage results in longer life with activity limitations in women (Table 2). A positive value of the disability effect in case of the gender difference in ULY means that the higher prevalence of activity limitations in women results in a longer life of women with activity limitations. The value of the disability effect is the same for the gender gap in HLY or in ULY, but the sign is reversed. In all countries but Austria and Estonia, both the ULY mortality and disability effect contribute to the fact that women are living more years with activity limitations. The mortality effect is positive in all countries [median ULY mortality effect: 3.4 years (IQR 2.5, range 5.9, Fig. 1)]. In all but two countries (Austria and Estonia), women also live more years with activity limitations because they have a higher prevalence of activity limitations.

High LE in either females or males, is associated with a reduction of the gender difference in ULY mainly since women’s mortality advantage is smaller [e.g., the coefficient_women’s LE_ is −0.49 or a 1-year increase in women’s LE is associated with a decrease in the mortality effect of the gender difference in ULY by almost 0.5 years (Table 3b)]. When the gender gap in LE is wide the gender gap in ULY is large mainly because of women’s larger mortality advantage (coefficient 0.70; Table 3b). The associations between the disability effect of the gender gaps in ULY and the LE indicators were not statistically significant. In populations with better health, there is a shift of the age groups contributing to the mortality effect of the gender difference in ULY towards older ages [coefficient_women’s LE_ is 2.03: the percent contribution of the age groups 50+ years to the mortality effect of the gender difference in ULY increases with 2 % when the LE in women is 1 year higher (Table 4b)]. When the gender difference in either LE, HLY or ULY is larger, a smaller part of the mortality effect on the gender difference in ULY can be attributed to difference in mortality at older ages (coefficient_gender difference LE_ is −1.98 %, Table 4b).

## Discussion

In this paper, we approach the health–survival paradox by using composite indicators, HLY and ULY, which contain information on both components of the paradox: mortality and the prevalence of (ill)-health. Mortality and disability tend to play in opposite ways on the magnitude of the gender differentials in HLY. While women’s longer life and higher disability prevalence translate into more years to be lived with disability by women, in all but two countries the disability effect compensates the mortality effect reducing the gender difference in HLY. Even more, in some countries, the disability effect overpasses the mortality effect and women live fewer years without disability. The health–survival paradox appears to be a function of the level of population health indicators and their gender difference. We observed that in populations with a high LE, the gender difference in HLY is small or even negative. Current cross-sectional analysis does not recognize that the health trajectories and the evolution of the LE during the last decades of the twentieth century were substantially different in Western European countries compared to countries of Central and Eastern Europe or the Baltic States (Leon 2011). Therefore, it is important to stress that a small or negative gender difference in HLY may mask important evolutions in the gender differences in mortality and/or activity limitations as it is a result of two opposing forces of the paradox: the survival with a smaller women’s mortality advantage and the health part with a larger women’s disability disadvantage. This observation is consistent with reports on trends in health expectancy indicators over time where more often evidence for compression of morbidity is reported among men alongside evidence for expansion among women (Robine et al. 2005; Van Oyen et al. 2008). The differences in evolution by gender in populations with a high LE may result from women having already reached extreme older ages and that changes in health are much more concentrated at the frontier of human life span. In populations with low life expectancy, we observed that the gender gap in HLY or ULY is large predominantly because of the large gender difference in mortality with the gender difference in the prevalence of activity limitations being less important. In populations with less favourable population health indicators such as low LE, low HLY and high ULY, the hardship among men is already evident at young ages (15–49 years), with men having higher mortality alongside a prevalence of activity limitations which is closer to that of women, yielding both an important mortality disadvantage and a lack of disability advantage relative to women. This confirms the double burden on men living in less healthy populations (countries of Central and Eastern Europe or the Baltic States) since together with their shorter life, they also have a shorter healthy life and a longer unhealthy life with a poorer health and higher mortality starting at young ages (Nusselder et al. 2010b).

Our analysis has several strengths. The country data were not pooled and the substantial heterogeneity in HLY and ULY among the EU member states is used (Jagger et al. 2008). The uncertainty around the estimates is accounted for in the meta-regression. The gender difference in HLY and ULY were subsequently separated into two parts to disentangle the health–survival paradox: one that can be explained by a differential age-specific mortality selection and the other that is due to a different age-specific prevalence of activity limitations.

Limitations of the study that should be considered are related to the cross-sectional design and the Sullivan method. The latter produces health expectancy indicators which are not period indicators and which may introduce bias in the absence of a steady state (Barendregt et al. 1994; Mathers and Robine 1997). The decomposition components do not represent the underlying processes of the incidence and recovery of activity limitations (Nusselder and Looman 2004). Further, the SILC survey is limited to the community dwelling population and no information is available on the health status of the institutionalized population. Not only does the proportion of the population within institutions differ between the EU countries, but the type of care-related institutions is also heterogeneous. Ignoring differences in health status between residents in the community and in institutions probably leads to an overestimation of the expected years without activity limitations. It is unknown if this bias occurs similarly in men and women in which case it would not affect gender differences. Even so, the bias may be larger in countries with a higher proportion of the population in institutions. A final limitation is related to the remaining problems in the harmonization of the GALI instrument (Van Oyen et al. 2010)

Multiple causes have been considered to explain the mechanisms which drive the health–survival paradox between the genders. The impact of methodological issues such as gender differences in survey participation or in reporting health problems have been minimized (Oksuzyan et al. 2009). Among the biological explanations most attention has been given to hormonal, autoimmune and genetic differences (Oksuzyan et al. 2008). The European LE experience during the most recent decades of last century suggests an impact of lifestyles and environmental factors including political, social and economic determinants of health, improvement of education, standards of living and health care (Leon 2011). Young men were more vulnerable to the negative health consequences of the rapid economic transition in the former communist states resulting in a high mortality of injuries, violence, cardiovascular diseases and cancers caused by high levels of alcohol consumption, especially binge drinking, smoking and poor nutrition (Mckee and Shkolnikov 2001). The lower quality of medical care in the Central and Eastern European Countries or the Baltic States may have further contributed to the more unfavourable health position of men (Newey et al. 2004). Both HLY and HLY gender gap are associated with country-specific macro-level indicators which are less favourable in countries of Central and Eastern Europe or the Baltic States (Jagger et al. 2008; Van Oyen et al. 2010). More specifically, the gender gap in HLY decreased as the gross domestic product, the expenditure on elderly care and the lifelong learning among men increased while it increased with a growing inequality in the income distribution (Van Oyen et al. 2010). Social position is an important determinant of inequality in health expectancy indictors (Bossuyt et al. 2004; Cambois et al. 2001; Crimmins and Saito 2001; Davis et al. 1999; Perenboom et al. 2005; Van Oyen et al. 2011). Women and especially older women have a lower social position as a result of a lower education or socio-economic position and this may affect the gender difference in health and functional disability (Bird and Rieker 2008). Several lifestyle factors, which have a differential uptake in men compared to women, not only affect LE, but are also associated with expected years of life without disability (Juel et al. 2008). Some of the lifestyle factors may especially influence mortality and reduce both the years lived with and without limitations; while other factors such as obesity mainly expand the years lived with disability (Reuser et al. 2009). Men have an excess of diseases which shorten life, while the disease pattern in women creates an excess in non-lethal conditions (NCHS 2009). Contributing causes of morbidity to the mortality effect of the gender difference in the disability-free life expectancy in the Netherlands were heart diseases, cancer, and COPD. Causes contributing to the disability effect of the gender difference in DFLE are heart disease, arthritis, back complaints, diabetes and COPD (Nusselder and Looman 2004). Within Europe, the wide range of gender differences in LE, HLY or ULY; the changing importance of either the mortality effect, the disability effect or the age groups contributing to the gender difference in HLY or ULY, is at the same time a statement that the health–survival paradox is not an artefact, but whatever determines the health–survival paradox is dependent on modifiable societal, social and behavioural factors.

The novelty of this paper studying the health–survival paradox is the use of HLY and the exploration of the two components of the gender difference in HLY: difference due to inequality in survival and difference due to inequality in disability. We observed large inequalities in the gender difference in health between European countries which corroborate our hypotheses. In populations with a high LE the gender difference in HLY is smaller because of the additive effect of a reduced mortality effect and a larger disability effect. In countries with a lower level of population health as indicated by a low LE, a low HLY and a large ULY, men are in the worst position having not only a higher mortality compared to women but also a high prevalence of activity limitations. Additionally, in contrast to men in populations with a better health profile, the ill-health of these men begins early in life.
